# The association between body roundness index and sarcopenia in older adults: a population-based study

**DOI:** 10.3389/fpubh.2025.1554491

**Published:** 2025-04-04

**Authors:** Jing Lyu, Zhiwu Liu, Hengjiang Gong, Tengfei Xu

**Affiliations:** ^1^The Department of General Practice, The First Hospital of Lanzhou University, Lanzhou, China; ^2^Geriatrics Ward 4, Department of Geriatrics, The First Hospital of Lanzhou University, Lanzhou, China; ^3^Medical Laboratory Center, The First Hospital of Lanzhou University, Lanzhou, China

**Keywords:** BRI, sarcopenia, older adults, NHANES, cross-sectional study

## Abstract

**Background:**

Sarcopenia, defined by the gradual decline in skeletal muscle mass and functionality, is a common disorder in the aging population and is linked to an elevated risk of falls and osteoporotic fractures. The contemporary diagnosis of sarcopenia depends on intricate and expensive techniques, such as computed tomography (CT) scans or dual-energy X-ray absorptiometry (DXA), which hinder the timely prevention of sarcopenia.

**Objective:**

This study seeks to explore the association between the Body Roundness Index (BRI) and sarcopenia in the older adult cohort, utilizing data from the National Health and Nutrition Examination Survey (NHANES) in the United States.

**Methods:**

Our study adopted a cross-sectional design, encompassing 9,411 older individuals, of which 1,147 were diagnosed with sarcopenia. After weighting, the number of individuals with sarcopenia was 23,985,011. The study employed multivariate logistic regression analysis to evaluate the association between BRI and sarcopenia, incorporating stepwise adjustments for potential confounders.

**Results:**

The outcomes of the multivariate logistic regression analysis revealed that, in contrast to individuals without sarcopenia, those with sarcopenia exhibited significantly higher mean BRI values and a greater prevalence of comorbid conditions, including hypertension and diabetes. A significant positive correlation was observed between BRI and the likelihood of developing sarcopenia. Specifically, after controlling for all covariates, each one-unit increase in BRI was linked to a 64% elevation in the risk of sarcopenia (OR = 1.64, 95% CI = 1.58–1.71). Furthermore, the receiver operating characteristic (ROC) curve analysis indicated that BRI is a robust predictor for diagnosing sarcopenia, with an AUC of 0.744.

**Conclusion:**

These findings suggest that, within the U.S. older adult population, an elevated BRI is associated with a heightened risk of sarcopenia. BRI can function as a practical and cost-effective anthropometric index for more precise prediction of sarcopenia risk in older adults.

## 1 Introduction

In recent years, growing public interest in health has led to significant advancements in understanding the prevalence and clinical relevance of abnormal body composition phenotype in clinical settings. Sarcopenia, a progressive disease characterized by the loss of skeletal muscle mass and quality, often results in declines in physical function (1, 2). Sarcopenia is particularly prevalent among the older adults, the reported prevalence of sarcopenia varies widely, ranging from 5% to 50% (3, 4), largely due to differences in diagnostic criteria, study populations, and assessment methods (5, 6). Furthermore, as global aging escalates, the incidence of sarcopenia is increasing (7). Prior research has demonstrated that sarcopenia elevates the risk of fractures and falls in the older adults, potentially leading to severe cases that may result in death (8). Additionally, studies suggest that the direct medical costs associated with sarcopenia total 18.5 billion dollars, and the medical costs for individuals with sarcopenia are higher compared to those without (9). Sarcopenia presents a major public health challenge for older adults, contributing to considerable economic strain on healthcare systems. This highlights the critical need for early identification and preventive measures among the aging population. At present, sarcopenia is primarily diagnosed using techniques like computed tomography (CT) or dual-energy X-ray absorptiometry (DXA), along with assessing physiological indicators such as grip strength and walking speed (10). Bioelectrical Impedance Analysis (BIA) is also a practical and non-invasive method for measuring body composition, which is easily affected by factors such as hydration status, environmental conditions, and individual differences. For example, it is sensitive to changes in hydration status and fluid balance, which can lead to inaccuracies in body composition estimates, particularly in individuals with altered fluid states like hypervolemia (11). However, in busy clinical settings, these diagnostic tools are often non-portable, complex to operate, and expensive, limiting their widespread adoption. Consequently, to identify and predict high-risk populations for sarcopenia at an early stage, identifying a direct, reliable, easily detectable, and cost-effective marker is crucial.

In 2013, Thomas et al. introduced the Body Roundness Index (BRI), a metric associated with obesity (12). It combines height (cm) and waist circumference (cm) to describe an individual's body shape. In contrast to traditional measures like Body Mass Index (BMI), waist circumference (WC), and hip circumference, BRI provides a more accurate representation of body fat and visceral fat proportions (13). A wealth of research has demonstrated that the Body Roundness Index (BRI) is strongly linked to age-related conditions, including coronary artery disease, carotid atherosclerosis, and diabetes (14–18). In addition, studies have shown that a significant proportion of older individuals with sarcopenia also have obesity. Sarcopenic obesity poses a serious health risk to the older adults (19). Early identification and prevention of sarcopenia and sarcopenic obesity are highly beneficial for the older adults. However, there has been no research exploring the connection between BRI and sarcopenia in the older adults population. Hence, this study seeks to investigate the relationship between BRI and sarcopenia in older adults by utilizing NHANES data.

## 2 Method

### 2.1 Study design and population

**Figure 1:**
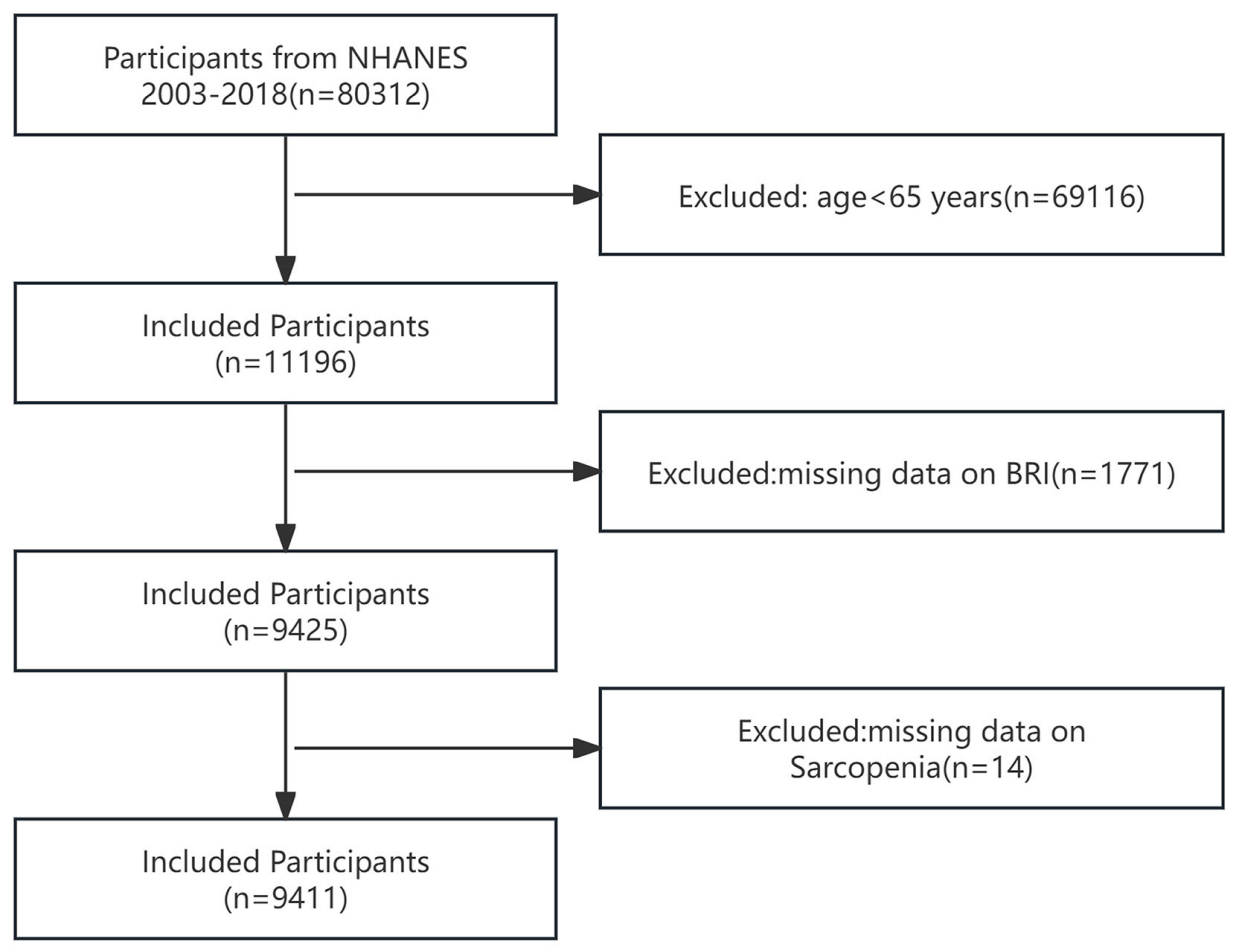
Flowchart for inclusion of study participants.

The NHANES database, overseen by the U.S. Centers for Disease Control and Prevention (CDC), is a national cross-sectional study aimed at evaluating the health and nutritional status of non-hospitalized residents in the United States (20). NHANES gathers extensive data on nutritional intake, health conditions, lifestyle factors, and various other aspects of both adults and children in the United States. Researchers can access the survey data online. The research protocol has received approval from the Ethics Review Board of the National Center for Health Statistics (NCHS) (20). Eligible researchers can access the database without requiring a formal application. To safeguard patient privacy, confidentiality protocols have been established to maintain the anonymity of all personal data. Additionally, participants have provided informed consent. Data from NHANES spanning sixteen consecutive years, from 2003 to 2018, were included, with a total of 80,312 participants. From 2003 to 2018, each wave spans 2 years. The periods 2003–2004, 2005–2006, 2007–2008, 2009–2010, 2011–2012, 2013–2014, 2015–2016, and 2017–2018 correspond to wave 1 through wave 8, respectively. According to the U.S. definition of the older adult, participants under the age of 65 were initially excluded, as they do not fall within the older adult population. Subsequently, participants lacking waist circumference or height data were excluded, as BRI could not be calculated without these data. Moreover, participants without sarcopenia data were excluded from the study. As a result, the final sample comprised 9,411 individuals. Among the 9,411 study participants, the final distribution of the included study population across waves was as follows: wave 1 included 1,232 cases (13.09%), wave 2 included 1,001 cases (10.64%), wave 3 included 1,323 cases (14.06%), wave 4 included 1,316 cases (13.98%), wave 5 included 1,015 cases (10.79%), wave 6 included 1,122 cases (11.92%), wave 7 included 1,147 cases (12.19%), and wave 8 included 1,255 cases (13.34%). The comprehensive screening procedure is depicted in Figure 1.

### 2.2 Definition of the BRI and sarcopenia

The formula for calculating BRI is (12):


$$\begin{array}{l} \text{BRI}=364.2-365.5\times \sqrt{1-\;{\left(\text{WC}/2\Pi \right)}^{2}/{0.5\text{Height}}^{2}} \end{array}$$

Waist circumference (WC) refers to the measurement around the waist, while height denotes the individual's body stature. All measurements were conducted at mobile examination centers, with both waist circumference and height recorded in centimeters.

Sarcopenia, as defined by the National Institutes of Health guidelines, is characterized by the ratio of Appendicular Skeletal Muscle Mass (ASM) to Body Mass Index (BMI). For females, a value below 0.512 indicates sarcopenia, while for males, a value below 0.789 indicates sarcopenia (21). Previous research has demonstrated that skeletal muscle mass can be estimated using factors such as height, weight, gender, and age (15). The specific calculation method for skeletal muscle mass is as follows: ASM = 0.193 × weight + 0.107 × height – 4.157 × gender – 0.037 × age – 2.63, Where Weight is in kilograms, Height is in centimeters, Gender is coded as 2 for females and 1 for males, Age is in years.

### 2.3 Assessment of covariates

The demographic covariates included in this study are age, gender, race, education level, marital status, and the poverty income ratio (PIR). Lifestyle covariates include smoking, alcohol, physical activity and dietary habits. Physical activity is assessed using the Metabolic Equivalent of Task (MET) metric. An activity level below 600 MET-min/weekly is classified as low activity, whereas a physical activity level of 600 MET-min/weekly or higher is classified as adequate. Dietary habits are assessed using the HEI-2015 (Healthy Eating Index-2015) score, where a higher score indicates a healthier diet. Comorbidity covariates include hypertension and diabetes. Laboratory test covariates in this study comprise total cholesterol (Tc), high-density lipoprotein (HDL), and glycated hemoglobin (HbA1c). Additionally, body mass index (BMI) is included as a covariate. The definitions of comorbidities and lifestyle factors are based on self-reports from respondents. A thorough analysis of these covariates enhances the understanding and interpretation of the study results.

### 2.4 Statistical analysis

The analysis included measures such as mean, median, standard deviation, range, and quartiles for continuous variables and frequency tables for categorical variables. Non-normally distributed continuous variables were summarized using the median and interquartile range (IQR). The analysis was conducted using complex sampling weights to ensure representatives of the study population. The survey package in R was utilized for this purpose, employing functions such as svymean(), svyvar(), and svyquantile() to calculate means, standard deviations, medians, and quartiles. To assess differences in variable characteristics across groups, we utilized the *t*-test and the survey Wilcoxon rank-sum test for continuous variables, accounting for complex survey samples. For categorical variables, we applied the Rao-Scott chi-squared test to analyze weighted percentages, ensuring a comprehensive description of the entire population.

In order to explore the association between BRI and sarcopenia in older adults, the Logistic regression models were employed. Multiple models were constructed, each adjusting for a different set of covariates to provide a nuanced understanding of how these covariates influence the observed association. Model 1 provided an unadjusted estimate, with no covariates included. In Model 2, adjustments were made for age, gender, race, education, marry status, and PIR to account for potential confounding factors. In Model 3, adjustments were made for age, gender, race, education level, marital status, poverty income ratio (PIR), body mass index (BMI), hypertension, diabetes, total cholesterol (Tc), high-density lipoprotein (HDL), glycated hemoglobin (HbA1c), smoking, and alcohol consumption to control for potential confounding factors.

Besides, data on the outcome variable, sarcopenia, and the continuous predictor variable, Body Roundness Index (BRI), were collected. To explore potential non-linear relationships between changes in BRI and sarcopenia, a logistic regression model with restricted cubic splines (RCS) was employed. Knots were tested at values between the 3rd and 7th percentiles, with the model yielding the lowest Akaike Information Criterion (AIC) selected for the RCS analysis. In this analysis, three knots were positioned at the 10th, 50th, and 90th percentiles.

Furthermore, the Receiver Operating Characteristic (ROC) curve was used to assess the predictive ability of the Body Roundness Index (BRI) for sarcopenia and to compare its performance with that of Body Mass Index (BMI). All statistical analyses in this study were conducted using R software (version 4.2.2). A two-sided *P*-value of < 0.05 was considered statistically significant for all tests.

## 3 Results

### 3.1 Baseline characteristics of the participants

The baseline characteristics table (Table 1) provides valuable insights into the demographic and clinical profile of the study population. Among individuals without sarcopenia, the weighted mean age was 72.7 years (SD = 5.5), whereas those with sarcopenia had a slightly higher mean age of 75.0 years (SD = 5.5), with this difference being statistically significant (*p* < 0.001). The average BRI was 5.79 7ra.92 in patients without sarcopenia and 7.63 6co.26 in patients with sarcopenia, inflecting a significant difference (*p* < 0.001). The gender distribution revealed that 44.7% of individuals without sarcopenia were male, whereas 46.5% of those in the sarcopenia group were male. However, this difference was not statistically significant (*p* = 0.384). Moreover, the prevalence of certain racial categories varied significantly between the two groups, with a higher proportion of non-Hispanic Whites in the group without sarcopenia (81.4%) compared to the sarcopenia group (65.4%) (*p* < 0.001). Significant differences were also observed in education level, marital status, physical activity, dietary habit, smoking habits, alcohol consumption, and the presence of comorbidities such as hypertension and diabetes. These findings highlight the importance of considering these baseline characteristics when exploring the relationship between sarcopenia and other health outcomes. In addition, the average BMI in the sarcopenia population was 31.8, with a median of 30.8. We conducted an additional analysis on the sarcoenic obesity population to observe the relationship between sarcopenic obesity and non-sarcopenic obesity. We found that the BRI of the sarcopenic obesity group was significantly higher than that of the non-sarcopenic obesity group (*p* < 0.001), and the difference was statistically significant (Supplementary Table 1).

**Table 1:** Weighted Patient demographics and baseline characteristics.

**Characteristic**	**Non-sarcopenia Weighted *N* = 254,421,263 Unweighted *n* = 8,264^a^**	**Sarcopenia Weighted *N* = 23,985,011 Unweighted *n* = 1,147^b^**	***p*-value**
**Age**			<0.001^b^
Mean ± SD	72.7 ± 5.5	75.0 ± 5.5	
Median (IQR)	72.0 (68.0, 78.0)	76.0 (70.0, 80.0)	
Range	65.0, 85.0	65.0, 85.0	
**Gender**			0.405^c^
male	44.7%	46.5%	
female	55.3%	53.5%	
**Race**			<0.001^c^
Mexican American	3.0%	10.5%	
Non-Hispanic Black	8.1%	5.6%	
Non-Hispanic White	81.4%	65.4%	
Other Hispanic	2.6%	9.2%	
Other Race	4.9%	9.4%	
**Education**			<0.001^c^
<9th Grade	8.3%	22.8%	
9–11th Grade	11.2%	16.9%	
High School Grad/GED or Equivalent	26.0%	24.2%	
Some College or AA degree	27.3%	24.3%	
College Graduate or above	27.1%	11.6%	
Unknown	0.1%	0.2%	
**Marry**			<0.001^c^
Married/Living with partner	62.4%	52.8%	
Widowed/Divorced/Separated/Never married	37.6%	47.1%	
Unknown	0.0%	0.1%	
**PIR**			<0.001^b^
Mean ± SD	2.93 ± 1.46	2.35 ± 1.31	
Median (IQR)	2.67 (1.71, 4.47)	2.15 (1.27, 3.16)	
Range	0.00, 5.00	0.00, 5.00	
**Physical activity**			<0.001^b^
Low (<600 MET-min/weekly)	53.1%	65.3%	
Moderate or higher (≥600 MET-min/weekly)	46.9%	34.7%	
**Dietary habits (HEI-2015)**			0.028^b^
Mean ± SD	55 ± 14	53 ± 14	
Median (IQR)	54 (45, 64)	53 (43, 63)	
Range	10, 96	15, 94	
**Smoking**			<0.001^c^
Yes	52.0%	42.7%	
No	47.9%	57.1%	
Unknown	0.1%	0.3%	
**Alcohol**			<0.001^c^
Yes	71.1%	60.3%	
No	28.8%	39.6%	
Unknown	0.1%	0.1%	
**Diabetes**			<0.001^c^
Yes	20.0%	25.4%	
Borderline	3.2%	4.2%	
No	76.9%	70.1%	
Unknown	0.0%	0.3%	
**Hypertension**			<0.001^c^
Yes	59.5%	68.6%	
No	40.3%	31.1%	
Unknown	0.2%	0.2%	
**ASM**			<0.001^b^
Mean ± SD	21.2 ± 5.4	19.5 ± 6.6	
Median (IQR)	20.8 (16.8, 25.2)	18.7 (14.0, 24.2)	
Range	7.5, 43.2	7.1, 41.6	
**BMI**			<0.001^b^
Mean ± SD	28.3 ± 5.5	31.8 ± 6.8	
Median (IQR)	27.6 (24.5, 31.4)	30.8 (27.3, 35.4)	
Range	13.2, 62.2	17.2, 63.6	
**BRI**			<0.001^b^
Mean ± SD	5.79 ± 1.92	7.63 ± 2.26	
Median (IQR)	5.56 (4.44, 6.89)	7.25 (6.05, 8.93)	
Range	1.19, 18.30	2.52, 17.74	
**Tc**			0.004^b^
Mean ± SD	193 ± 42	189 ± 42	
Median (IQR)	191 (164, 219)	186 (160, 210)	
Range	83, 431	94, 350	
**HDL**			<0.001^b^
Mean ± SD	56 ± 17	53 ± 15	
Median (IQR)	54 (44, 66)	51 (41, 61)	
Range	11, 226	17, 126	
**HbA1c**			<0.001^b^
Mean ± SD	5.92 ± 0.87	6.10 ± 0.93	
Median (IQR)	5.70 (5.40, 6.10)	5.90 (5.60, 6.30)	
Range	2.00, 14.30	4.30, 13.20	

PIR, poverty income ratio; MET, Metabolic Equivalents Task; HEI-2015, Healthy eating index-2015; ASM, Appendicular Skeletal Muscle Mass; BMI, body mass index; BRI, Body roundness index; Tc, total cholesterol; HDL, High-density lipoprotein; HbA1c, Glycated hemoglobin.

a %.

b t-test adapted to complex survey samples.

c chi-squared test with Rao & Scott's second-order correction.

### 3.2 The associations between BRI and sarcopenia

In the logistic regression analysis examining the association between body roundness index (BRI) and the risk of sarcopenia, several models with varying covariates adjustments were evaluated. For BRI as a continuous variable, each unit increase was associated with a significant increase in the odds of sarcopenia across all models, with odds ratios (OR) escalating from 1.49 (95% confidence interval [CI]: 1.45–1.54, *p* < 0.001) in the crude model to 1.64 (95% CI: 1.96–2.33, *p* < 0.001) in the fully adjusted model. When BRI was categorized into quartiles, the analysis revealed a progressive increase in risk for higher quartiles compared to the reference group in each model. For instance, those in the highest quartile (Q4 [7.08, 18.3]) had notably increased odds of sarcopenia, particularly in Model 2 (OR: 15.53, 95% CI: 11.52–20.92, *p* < 0.001) and Model 3 (OR: 17.65, 95% CI: 12.96–24.04, *p* < 0.001). A significant trend (*p* < 0.001) was observed across all models, indicating a dose-response relationship between BRI and sarcopenia risk. These associations remained robust after adjusting for demographic, socioeconomic, and health-related covariates. For detailed data, please refer to Table 2.

**Table 2:** Association between BRI and sarcopenia (Logistic regression).

**Characteristic**	**Model 1**			**Model 2**			**Model 3**		
	**OR** ^a^	**95% CI** ^a^	***p*** **-value**	**OR** ^a^	**95% CI** ^a^	***p*** **-value**	**OR** ^a^	**95% CI** ^a^	***p*** **-value**
**BRI (continuous)**	1.49	1.45, 1.54	<0.001	1.60	1.55, 1.66	<0.001	1.64	1.58, 1.71	<0.001
**BRI**									
Q1 [1.19, 4.56)	–	–		–	–		–	–	
Q2 [4.56,5.74)	2.45	1.82, 3.28	<0.001	2.43	1.75, 3.37	<0.001	2.54	1.83, 3.54	<0.001
Q3 [5.74,7.08)	5.34	4.07, 7.01	<0.001	5.58	4.11, 7.57	<0.001	6.06	4.44, 8.27	<0.001
Q4 [7.08,18.3]	12.17	9.37, 15.80	<0.001	15.53	11.52, 20.92	<0.001	17.65	12.96, 24.04	<0.001
P for trend			<0.001			<0.001			<0.001

a OR, Odds Ratio; CI, Confidence Interval.

Model 1: no covariates were adjusted; Model 2: adjusted for age, gender, race, education, marry status, PIR, physical activity, dietary habit; Model 3: adjusted for age, gender, race, education, marry status, PIR, physical activity, dietary habit, Hypertension, Diabetes, Tc, HDL, HbA1c, Smoking, and Alcohol.

### 3.3 Subgroup analysis

In Table 3, the subgroup analysis revealed statistically significant associations between BRI and sarcopenia across all age groups. In the overall population (N = 9,411), the odds ratio (OR) was 1.49 (95% CI: 1.45–1.54, P < 0.001). Upon age stratification, the odds of sarcopenia remained consistently elevated, with ORs of 1.51 (95% CI: 1.45–1.57, P < 0.001) for individuals aged <75 years (N = 5,263), 1.53 (95% CI: 1.46–1.61, P < 0.001) for those aged 75–85 years (N = 3,889), and 2.02 (95% CI: 1.55–2.63, P < 0.001) for individuals aged ≥85 years (N = 259). The interaction analysis by age group showed no statistically significant interaction effect (P for interaction = 0.076). These findings suggest that BRI is a robust predictor of sarcopenia across various age cohorts.

**Table 3:** Associations between age and sarcopenia in the stratified analysis.

**Subgroup**	**N**	**Crude OR (95% CI)**	**P-value**	**P for interaction**
Overall	9,411	1.49 (1.45–1.54)	<0.001	
Age group				0.076
<75	5,263	1.51 (1.45–1.57)	<0.001	
75–85	3,889	1.53 (1.46–1.61)	<0.001	
≥85	259	2.02 (1.55–2.63)	<0.001	

### 3.4 Non-linear association between the BRI and sarcopenia

**Figure 2:**
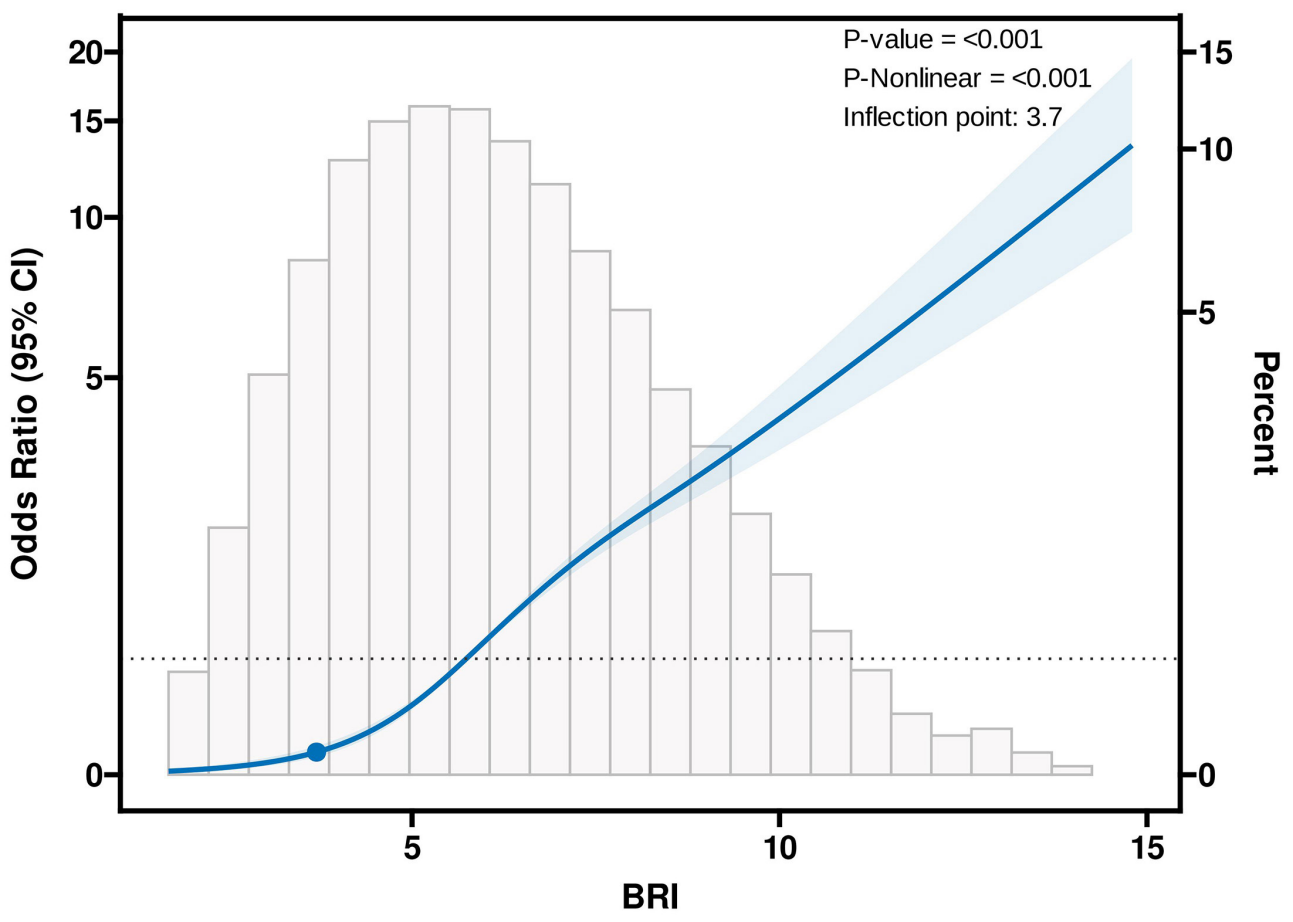
Association between BRI and sarcopenia with the RCS function. Model with 3 knots located at 10th, 50th, and 90th percentiles. Y-axis represents the OR to present sarcopenia for any value of BRI compared to individuals with reference value (50th percentile) of BRI.

The RCS analysis indicated a non-linear relationship between BRI and sarcopenia. The inflection point of the RCS curve was identified at a BRI value of 3.7, marking a critical threshold in the association between BRI and sarcopenia (Figure 2). Based on this inflection point, the data were divided into two groups: BRI < 3.7 and BRI ≥ 3.7. Segmented regression was conducted separately for each group, and the results, presented in Table 4, demonstrate that the effect of BRI on sarcopenia varies across different ranges. When BRI is ≥3.7, its association with sarcopenia is stronger and statistically highly significant (*p* < 0.001). In contrast, when BRI is <3.7, the association is relatively strong, but it does not reach statistical significance (*p* = 0.051).

**Table 4:** Effect of standardized BRI level on sarcopenia: odds ratios from segmented logistic regression analysis.

**Characteristic**	**OR per SD^a^**	**95% CI^a^**	***p*-value**
BRI (<3.7)	2.31	1.0, 5.36	0.051
BRI (≥3.7)	1.99	1.88, 2.11	<0.001

a OR, Odds Ratio; CI, Confidence Interval.

### 3.5 The predictive capacity of BRI for sarcopenia

**Figure 3:**
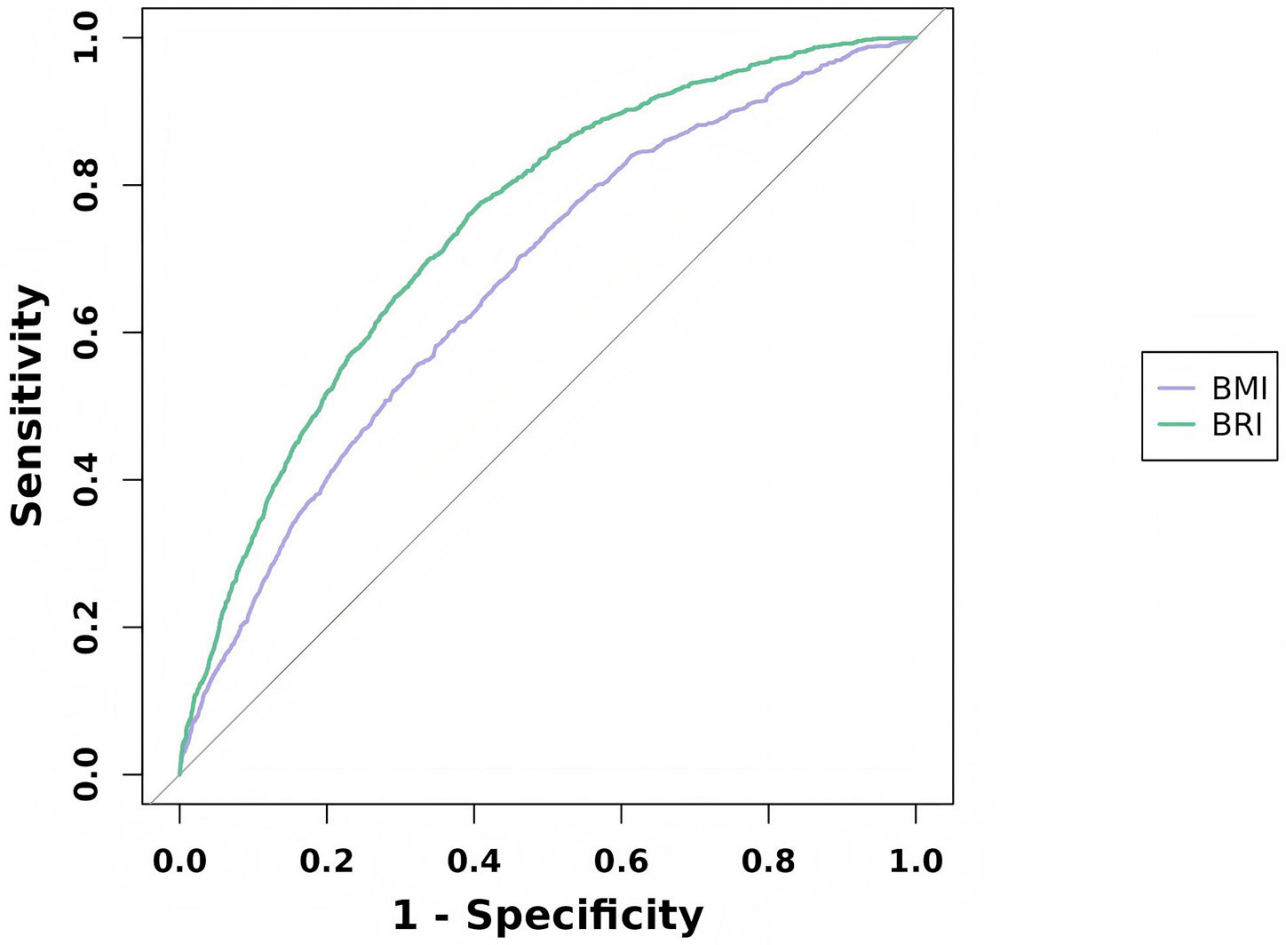
ROC curve for sarcopenia.

Figure 3 displays the ROC curves illustrating the predictive capability of BRI and BMI for sarcopenia. The AUC of BRI was 0.744 (95% CI 0.729–0.758), The AUC of BMI was 0.666 (95% CI 0.650–0.682), Compared to BMI, BRI has better performance in predicting and diagnosing sarcopenia. Using Youden's Index, the optimal cutoff value for diagnosing sarcopenia with BRI was determined to be 6.01, this analysis resulted in a sensitivity of 76.00% and a specificity of 60.80%. BRI demonstrated excellent predictive ability for sarcopenia.

## 4 Discussion

The relationship between BRI and sarcopenia was investigated using the NHANES dataset, which included 9,411 individuals. Prior to weighting, 1,147 individuals with sarcopenia and 8,264 without were identified, resulting in a prevalence of 12.2%, consistent with findings from related research (22). To further examine this, calculations were performed based on NHANES weighting standards, indicating a total population of 278,406,274, with 23,589,011 individuals identified as having sarcopenia. The weighted prevalence of sarcopenia was approximately 8.5%, lower than the unweighted calculation, likely due to the larger population sample. With a broader population base, the prevalence may more accurately reflect the actual situation. In our study, we found that the proportion of individuals with low physical activity was significantly higher among those with sarcopenia than among individuals with normal physical activity levels, suggesting that low physical activity may be a contributing factor to the development of sarcopenia. We also observed that individuals with sarcopenia had a lower dietary health index compared to non-sarcopenic individuals, indicating that healthy eating habits play a positive role in preventing sarcopenia. Notably, we observed that the proportion of smokers was higher among individuals with sarcopenia than among non-sarcopenic individuals, which contradicts the commonly held belief that smoking is a risk factor for sarcopenia. This discrepancy may be related to variability in the study population characteristics and the sample size. Multivariate logistic regression analysis revealed a significant positive correlation between BRI and sarcopenia. This association remained strong and consistent even after adjusting for relevant covariates, such as gender, age, ethnicity, and other relevant factors. Furthermore, the RCS analysis revealed a non-linear relationship between BRI and sarcopenia. The ROC curve also demonstrated that BRI has strong predictive value for the occurrence of sarcopenia, outperforming BMI. These findings suggest that BRI may serve as a valuable clinical indicator for the early detection of sarcopenia in the older adult population, and that regular monitoring of BRI holds significant clinical value for early intervention in sarcopenia cases.

In this study, we observed that individuals with sarcopenia exhibited significantly higher BMI values than their non-sarcopenia counterparts. The average BMI in the sarcopenic group was 31.8 (with a median of 30.8), indicating that sarcopenia should be closely monitored in the older adult population with elevated BMI values. Furthermore, using the World Health Organization's diagnostic criteria for obesity, we categorized the sarcopenic study population into two groups: sarcopenic obesity and non-sarcopenic obesity. Among the 1,147 sarcopenia individuals, 626 (54.57%) were classified as having sarcopenic obesity, while 521 were categorized as non-sarcopenic obesity. This highlights a high prevalence of obesity (54.57%) among older adults with sarcopenia. Given that obesity is a risk factor for numerous chronic diseases, targeted attention should be directed toward obesity management in this demographic. Additionally, the study found that the Body Roundness Index (BRI) was significantly higher in individuals with sarcopenic obesity compared to those with non-sarcopenic obesity, suggesting that BRI could serve as an effective clinical indicator for the early detection of sarcopenic obesity.

To the best of our knowledge, this is the first study to explore the relationship between BRI and sarcopenia in the older adult population. Previous research has shown that BRI is associated with a variety of diseases, including cardiovascular diseases, respiratory diseases, diabetes, fatty liver, obesity, osteoarticular diseases, mental health disorders, and cancer (23–25). These findings provide a theoretical foundation for using BRI as a tool for assessing health. Our study identified an increased BRI as a risk factor for sarcopenia in the older adults, with BRI being a more effective predictor of sarcopenia occurrence than BMI in this population. While the precise mechanisms underlying the relationship between BRI and sarcopenia in the older adults require further investigation, several potential explanations have been proposed. BRI is a novel anthropometric index developed to more accurately represent an individual's body shape and fat distribution (26). The calculation of BRI places particular emphasis on waist circumference, which, in contrast to traditional BMI, more accurately reflects abdominal fat distribution and indicates the accumulation of visceral fat and associated metabolic risks. As a result, BRI is linked to adipose tissue accumulation and metabolic disorders (27). The higher the BRI value, the more severe central obesity is, which means more accumulation of visceral fat. Adipose tissue serves not only as an energy store but also as an active endocrine organ capable of releasing inflammatory cytokines (28), such as TNF-α and interleukin-6 (IL-6), which can contribute to muscle breakdown, inhibit muscle synthesis, induce muscle atrophy, and ultimately lead to sarcopenia (29). Studies have shown that interleukin-6 (IL-6) primarily accelerates muscle loss by inhibiting muscle protein synthesis, enhancing protein degradation, inducing insulin resistance, and suppressing muscle regeneration. IL-6 inhibits mTOR through the JAK-STAT3 signaling pathway, thereby reducing protein synthesis while concurrently activating NF-κB and FoxO3a, which promote the ubiquitin-proteasome system (UPS) and the autophagy-lysosome pathway (ALP), leading to accelerated muscle protein degradation. Additionally, IL-6 induces SOCS3-mediated insulin resistance, resulting in a diminished energy supply to muscles and facilitating the accumulation of free fatty acids (FFA), which further exacerbates muscle damage. Persistently elevated IL-6 levels further impair muscle repair and regeneration by inhibiting satellite cell proliferation and promoting muscle fibrosis (30–33). Similarly, research has demonstrated that TNF-α accelerates muscle protein degradation by activating the NF-κB signaling pathway, upregulating the ubiquitin-proteasome system (UPS) and the autophagy-lysosome pathway, while concurrently inhibiting the Akt/mTOR signaling pathway, culminating in decreased muscle protein synthesis. Additionally, TNF-α induces muscle cell apoptosis via the Fas receptor and mitochondrial pathways and promotes the release of inflammatory cytokines IL-6 and IL-1β, contributing to a sustained inflammatory state, which exacerbates muscle dysfunction. Moreover, TNF-α promotes the loss of type II fast-twitch muscle fibers, ultimately resulting in decreased muscle strength (34–36). Furthermore, the continued expansion of adipose tissue may lead to obesity, with research suggesting that obesity and sarcopenia can coexist due to factors such as malnutrition, inflammation, and insulin resistance (37). Insulin, a crucial hormone for muscle growth, may be suppressed by insulin resistance, in the state of insulin resistance (IR), the PI3K-Akt-mTOR pathway is impaired, leading to reduced protein synthesis and increased degradation. Meanwhile, abnormalities in glucose metabolism restrict energy supply to muscles, ultimately promoting muscle deterioration and accelerating muscle atrophy (38–40). Finally, obesity not only impacts body composition but also correlates with functional decline, such as gait abnormalities and an increased risk of falls, which further exacerbate sarcopenia development. In conclusion, the elevated fat tissue indicated by an increased BRI, which may lead to obesity, can contribute to muscle degeneration through various mechanisms, including chronic inflammation, insulin resistance, and disruptions in fat metabolism, ultimately affecting muscle mass and function. Therefore, older individuals with elevated BRI levels should receive focused attention for the early prevention, intervention, and management of sarcopenia. Our study provides several notable advantages. Firstly, as previously mentioned, this study is the first to establish a relationship between BRI and sarcopenia in the older adult population, as documented in published research. Additionally, the non-linear relationship between BRI and the risk of sarcopenia was identified through multiple regression analysis and RCS curve analysis. These findings contribute to a deeper understanding of the association between BRI and sarcopenia. Lastly, this study is based on the NHANES database, which ensures the objectivity of the information. The sample is highly representative, owing to its multi-ethnic composition, and the large sample size further strengthens the stability and generalizability of the results. Therefore, in clinical practice, BRI serves as a simple and effective assessment tool capable of rapidly indicating trends in abdominal obesity among patients. It can help clinicians implement intervention strategies, such as improving dietary habits and encouraging physical exercise, to reduce the risk of sarcopenia.

However, this study also has several limitations. Firstly, due to the cross-sectional design of the study, the relationship between BRI and sarcopenia cannot be definitively determined. Secondly, this study, as it utilizes data from the NHANES database, did not analyze the relationship between BRI and traditional sarcopenia prediction indicators such as calf circumference, nor did it assess whether BRI can supplement the predictive ability of calf circumference when used alone for sarcopenia prediction. Therefore, further prospective studies with larger sample sizes are necessary to clarify the causal relationship. In addition, despite adjusting for numerous potential covariates, the effects of other potential confounding factors cannot be entirely excluded. Lastly, different countries and regions utilize distinct diagnostic criteria for sarcopenia. Since the data used in this study were sourced from a public database in the United States, the diagnostic criteria for sarcopenia established by the National Institutes of Health were strictly followed. Variations in diagnostic criteria across regions may influence the correlation between BRI and sarcopenia.

## 5 Conclusion

Our study suggests that a higher BRI is associated with an increased risk of sarcopenia in the older adult population in the United States. BRI may serve as a useful anthropometric index for more precise prediction of sarcopenia risk in older adults. Future research should explore the underlying mechanisms and potential benefits of BRI in relation to sarcopenia through basic research and prospective cohort studies.

## Data Availability

The raw data supporting the conclusions of this article will be made available by the authors, without undue reservation.
