# Supplementary material for: The association between body roundness index and sarcopenia in older adults: a population-based study
Source: Front Public Health. 2025 Apr 4;13:1554491. doi: 10.3389/fpubh.2025.1554491 (PMC12006143; doi:10.3389/fpubh.2025.1554491)
Supplement: Supplementary file 1 [file Table_1.docx]

Supplementary Table 1

| **Characteristic** | **Non sarcopenic obesity**  **N = 521^1^** | **Sarcopenic obesity**  **N = 626^1^** | **p-value** |
| --- | --- | --- | --- |
| **Age** |  |  | <0.001^2^ |
| Mean ± SD | 76.3 ± 5.5 | 73.5 ± 5.3 |  |
| Median (IQR) | 79.0 (72.0, 80.0) | 73.0 (69.0, 79.0) |  |
| Range | 65.0, 85.0 | 65.0, 85.0 |  |
| **Gender** |  |  | <0.001^3^ |
| female | 300 (57.6%) | 278 (44.4%) |  |
| male | 221 (42.4%) | 348 (55.6%) |  |
| **Race** |  |  | <0.001^3^ |
| Mexican American | 115 (22.1%) | 154 (24.6%) |  |
| Non-Hispanic White | 206 (39.5%) | 278 (44.4%) |  |
| Non-Hispanic Black | 34 (6.5%) | 69 (11.0%) |  |
| Other Hispanic | 80 (15.4%) | 91 (14.5%) |  |
| Other Race | 86 (16.5%) | 34 (5.4%) |  |
| **Education** |  |  | 0.071^3^ |
| Less Than 9th Grade | 197 (37.8%) | 211 (33.7%) |  |
| 9-11th Grade | 87 (16.7%) | 111 (17.7%) |  |
| High School Grad/GED or Equivalent | 103 (19.8%) | 124 (19.8%) |  |
| College Graduate or above | 56 (10.7%) | 50 (8.0%) |  |
| Some College or AA degree | 76 (14.6%) | 127 (20.3%) |  |
| Unknown | 2 (0.4%) | 3 (0.5%) |  |
| **Marry** |  |  | <0.001^4^ |
| Widowed/Divorced/Separated/ Never married | 279 (53.6%) | 275 (43.9%) |  |
| Married/Living with partner | 241 (46.3%) | 351 (56.1%) |  |
| Unknown | 1 (0.2%) | 0 (0.0%) |  |
| **PIR** |  |  | 0.717^2^ |
| Mean ± SD | 2.00 ± 1.19 | 2.01 ± 1.27 |  |
| Median (IQR) | 1.78 (1.10, 2.62) | 1.67 (1.02, 2.72) |  |
| Range | 0.00, 5.00 | 0.00, 5.00 |  |
| **Smoking** |  |  | <0.001^4^ |
| Yes | 187 (35.9%) | 299 (47.8%) |  |
| No | 332 (63.7%) | 326 (52.1%) |  |
| Unknown | 2 (0.4%) | 1 (0.2%) |  |
| **Alcohol** |  |  | <0.001^4^ |
| Yes | 282 (54.1%) | 404 (64.5%) |  |
| No | 238 (45.7%) | 222 (35.5%) |  |
| Unknown | 1 (0.2%) | 0 (0.0%) |  |
| **Diabetes** |  |  | <0.001^4^ |
| Yes | 107 (20.5%) | 221 (35.3%) |  |
| Borderline | 16 (3.1%) | 29 (4.6%) |  |
| No | 397 (76.2%) | 373 (59.6%) |  |
| Unknown | 1 (0.2%) | 3 (0.5%) |  |
| **Hypertension** |  |  | 0.004^4^ |
| Yes | 326 (62.6%) | 444 (70.9%) |  |
| No | 194 (37.2%) | 181 (28.9%) |  |
| Unknown | 1 (0.2%) | 1 (0.2%) |  |
| **ASM** |  |  | <0.001^2^ |
| Mean ± SD | 15.8 ± 4.3 | 22.6 ± 5.8 |  |
| Median (IQR) | 14.1 (12.3, 20.2) | 23.4 (17.1, 26.6) |  |
| Range | 7.1, 23.5 | 10.1, 41.6 |  |
| **BMI** |  |  | <0.001^2^ |
| Mean ± SD | 26 ± 3 | 36 ± 5 |  |
| Median (IQR) | 27 (25, 28) | 35 (32, 38) |  |
| Range | 17, 30 | 30, 64 |  |
| **BRI** |  |  | <0.001^2^ |
| Mean ± SD | 5.94 ± 1.13 | 8.88 ± 1.89 |  |
| Median (IQR) | 5.98 (5.26, 6.66) | 8.55 (7.54, 9.76) |  |
| Range | 2.52, 9.33 | 4.98, 17.74 |  |
| **Tc** |  |  | 0.016^2^ |
| Mean ± SD | 192 ± 43 | 186 ± 41 |  |
| Median (IQR) | 189 (162, 212) | 184 (158, 209) |  |
| Range | 100, 350 | 94, 345 |  |
| **HDL** |  |  | <0.001^2^ |
| Mean ± SD | 54 ± 15 | 50 ± 14 |  |
| Median (IQR) | 53 (44, 62) | 48 (40, 58) |  |
| Range | 17, 126 | 22, 108 |  |
| **HbA1c** |  |  | <0.001^2^ |
| Mean ± SD | 6.01 ± 0.90 | 6.36 ± 1.13 |  |
| Median (IQR) | 5.80 (5.50, 6.20) | 6.00 (5.70, 6.70) |  |
| Range | 4.40, 13.10 | 4.30, 13.20 |  |
| ^1^n (%) | | | |
| ^2^Wilcoxon rank sum test | | | |
| ^3^Pearson's Chi-squared test | | | |
| ^4^Fisher's exact test | | | |
| Table 1: Patient demographics and baseline characteristics | | | |
